# Enzyme replacement therapy (ERT) in pompe disease

**DOI:** 10.1186/1824-7288-40-S1-A64

**Published:** 2014-08-11

**Authors:** Agata Fiumara

**Affiliations:** 1 Centro Riferimento Regionale per le Malattie Metaboliche, UO Clinica Pediatrica, Dip. Scienze Mediche e Pediatriche, Università di Catania, Italy

## 

Pompe disease (OMIM 232300) is an AR glycogenosis due to deficiency of the lysosomal enzyme alpha-glucosidase (GAA). As a result, glycogen storage occurs in muscles and patients present a wide clinical spectrum ranging from early onset severe cardiomyopathy (EOPD) to adult onset forms (LOPD). Severe loss of GAA activity correlates with early onset and severe phenotypes. Residual enzyme activity warrants later onset and a slowly progressive course, although a strict correlation is not always observed. Patients with Classic infantile Pompe disease show hypotonia, macroglossia, respiratory insufficiency and early death for cardiorespiratory failure.

The availability of enzyme replacement therapy (ERT) with alglucosidase alfa ( human recombinant GAA) has changed the natural history of the disease allowing most children with EOPD a longer survival and a longer preserved muscle performance with increased quality of life in LOPD.

The experience of the Regional referral Center for Metabolic Diseases, University of Catania, concerns 11 patients ( 9 months to 65 years at the time of the diagnosis). Their clinical and laboratory follow-up included eye tracking and Muscle MRI.

EOPD, with a severe clinical picture since the first weeks of life and cardiomyopathy was observed in a newborn who died at age 4 months, despite early started and high dosage ERT. Another girl with EOPD is on ERT since the age of 9 months and, today, after 7 years of treatment shows only mild signs of muscle weakness and no cardiac involvement.

Five more LOPD patients on ERT had a significant improvement of blood parameters including transaminases, LDH and CPK. No progression of muscle impairment was seen after 5 to 8 years of treatment, except for one 35 years old man, who was already severely compromised and did not show any benefit in terms of respiratory ventilation.

In a recently performed Italian survey of classical Pompe disease treated with ERT, a substantial improvement in cardiac parameters and gross motor function was observed, even if residual muscle weakness still exist and gross motor function remain below age-appropriate levels.

The different response to ERT depends on several factors mainly on timing of treatment (worse outcome if started later than 5 months of age) and cross reactive immune material (CRIM-negative status associated with less good response to ERT).

Although some residual mild weakness is present in treated patients, ERT has demonstrated its effects on respiration, lowering nocturnal apneas and allowing patients a better quality of life.

